# Role of the Pre-neck Appendage Protein (Dpo7) from Phage vB_SepiS-phiIPLA7 as an Anti-biofilm Agent in Staphylococcal Species

**DOI:** 10.3389/fmicb.2015.01315

**Published:** 2015-11-25

**Authors:** Diana Gutiérrez, Yves Briers, Lorena Rodríguez-Rubio, Beatriz Martínez, Ana Rodríguez, Rob Lavigne, Pilar García

**Affiliations:** ^1^Consejo Superior de Investigaciones Científicas – Instituto de Productos Lácteos de AsturiasVillaviciosa, Spain; ^2^Laboratory of Gene Technology, KU LeuvenHeverlee, Belgium; ^3^Laboratory of Applied Biotechnology, Ghent UniversityGhent, Belgium

**Keywords:** exopolysaccharide depolymerase, biofilm, biofilm matrix, *S. epidermidis*, *S. aureus*

## Abstract

*Staphylococcus epidermidis* and *Staphylococcus aureus* are important causative agents of hospital-acquired infections and bacteremia, likely due to their ability to form biofilms. The production of a dense exopolysaccharide (EPS) matrix enclosing the cells slows the penetration of antibiotic down, resulting in therapy failure. The EPS depolymerase (Dpo7) derived from bacteriophage vB_SepiS-phiIPLA7, was overexpressed in *Escherichia coli* and characterized. A dose dependent but time independent response was observed after treatment of staphylococcal 24 h-biofilms with Dpo7. Maximum removal (>90%) of biofilm-attached cells was obtained with 0.15 μM of Dpo7 in all polysaccharide producer strains but Dpo7 failed to eliminate polysaccharide-independent biofilm formed by *S. aureus* V329. Moreover, the pre-treatment of polystyrene surfaces with Dpo7 reduced the biofilm biomass by 53–85% in the 67% of the tested strains. This study supports the use of phage-encoded EPS depolymerases to prevent and disperse staphylococcal biofilms, thereby making bacteria more susceptible to the action of antimicrobials.

## Introduction

Biofilms are surface-attached microbial communities surrounded by a polymeric matrix, which protects them from the external environment. This complex structure confers to the bacteria a high resistance to antibiotics, disinfectants and to the host, an immune system response (Hall-Stoodley and Stoodley, 2009). The ability to develop a biofilm is one of the major virulence factors in many pathogenic bacteria, while it is a key factor for colonization in opportunistic bacteria. *Staphylococcus epidermidis* is a prominent example of a bacterial species with a pathogenicity associated to the biofilm formation on medical devices (Rupp, 2014), being the most common cause of bacteremia in immune-compromised patients (Rogers et al., 2009). In addition, methicillin resistant strains (MRSE) carrying the staphylococcal cassette chromosome *mec* (SCC*mec*), are widely spread within hospitals (Iorio et al., 2012), and provide a reservoir of resistance that might be transferred to *Staphylococcus aureus* (Otto, 2013), also a frequent cause of nosocomial infections. *S. aureus* encodes a number of virulence factors that enable to infect host tissues (Lowy, 1998). In addition, methicillin-resistant *S. aureus* (MRSA) isolates, resistant to all available penicillins and other β-lactam antibiotics, have rapidly disseminated beyond clinical settings among general population (David and Daum, 2010) and livestock (Price et al., 2012).

Extracellular material of staphylococcal biofilms is a complex combination of polysaccharides, teichoic acids, proteins and DNA. The polysaccharide intercellular adhesin (PIA/PNAG) is a poly-β-1,6-*N*-acetyl glucosamine, which production is dependent on the presence of the operon icaADBC (Mack et al., 1996). However, staphylococcal strains with a PIA/PNAG-independent biofilm were also identified. In this case, the extracellular matrix is based on the presence of adhesive proteins such as Aap, Embp, Bhp, and Bap (Hussain et al., 1997; Cucarella et al., 2001; Zhang et al., 2003; Christner et al., 2010). The presence of teichoic acids in staphylococcal species enhances adhesion of bacterial cells to fibronectin, increasing their pathogenicity (Hussain et al., 2001; Vergara-Irigaray et al., 2008). In addition, a number of microbial surface components recognizing adhesive matrix molecules (MSCRAMMs) are able to interact with the host matrix proteins (Patti et al., 1994). Finally, the modulation of the extracellular genomic DNA (eDNA) release and degradation was shown as an important factor in biofilm maturation (Mann et al., 2009).

Due to the importance of biofilms in hospital settings, several approaches to prevent and remove them have been assayed. New materials were designed by coating their surface with different antimicrobials, like bacteriocins and essential oils, which caused a reduction in biomass and viability of bacteria in biofilms (Nostro et al., 2010, 2013). Other changes in physicochemical properties of biomaterial surfaces resulted in reduction of bacterial adhesion and further biofilm formation (Tang et al., 2009). Additional strategies were focused on small molecules that interfere with the expression of virulence genes including those necessary for biofilm formation (Ma et al., 2012). Finally, it is noteworthy that previously formed biofilms can be removed by degrading the extracellular matrix or by killing the bacteria inside the structure. Alternatives to antibiotics for killing bacteria and biofilm removal include bacteriocins (Saising et al., 2012), bacteriophages (Cerca et al., 2007; Gutiérrez et al., 2012, 2015) and endolysins (Domenech et al., 2011; Shen et al., 2013; Gutiérrez et al., 2014). However, to eradicate the biofilm structure a successful degradation of extracellular matrix is required. Regarding this, PIA-degrading enzymes like dispersin B (Kaplan et al., 2004) and bacteriophage-encoded polysaccharide depolymerase proteins (Cornelissen et al., 2012) have been proposed.

We have previously identified and characterized two bacteriophages infecting *S. epidermidis*, vB_SepiS-phiIPLA5 and vB_SepiS-phiIPLA7, which exhibit plaques surrounded by an increasing halo zone indicative of the presence of polysaccharide depolymerase activity (Gutiérrez et al., 2010). Genomic characterization of phage vB_SepiS-phiIPLA7 showed a protein (gp18, 98.5 kDa) located in the structural region containing two catalytic domains. A putative pectin lyase domain was identified at the amino-terminal part of the protein, and a putative peptidase domain at the C-terminus. It was suggested that the anti-biofilm activity showed by phage vB_SepiS-phiIPLA7 may be attributed to this protein (Gutiérrez et al., 2012). In the present work, protein gp18 named Dpo7, was overexpressed in *E. coli* and purified. The polysaccharide depolymerase activity of Dpo7 was confirmed against *S. epidermidis* and *S. aureus* biofilms.

## Materials and Methods

### Bacterial Strains and Growth Conditions

Ten different *S. epidermidis* strains and two *S. aureus* strains were used in this study (**Table 1**). All bacteria were isolated in Baird–Parker (BP) agar and routinely cultured in TSB broth (Tryptic Soy Broth, Scharlau, Barcelona, Spain) at 37°C with shaking or in TSB plates containing 2% (w/v) bacteriological agar (TSA). *E. coli* transformants were selected on LB medium (1% tryptone, 0.5% yeast extract, 1% NaCl) supplemented with 2% (w/v) bacteriological agar and 100 μg ml^-1^ ampicillin at 37°C.

**Table 1:** Strains used in this study.

	Strain	Reference
*S. epidermidis*	F12	Delgado et al., 2009
	B	
	DH3LIK	
	YLIC13	
	Z2LDC14	
	DG2n	
	ASLD1	
	LO5081	
	LV5RB3	
	LO5RB1	
*S. aureus*	15981	Valle et al., 2003
	V329	Cucarella et al., 2001

### Cloning and Overexpression of Dpo7

The codon usage of the *orf18* gene, encoding Dpo7 (GenBank accession number YP_006561180.1) from phage vB_SepiS-phiIPLA7 was optimized based on *E. coli* codon usage by the OptimumGene^TM^ Codon Optimization Technology. Additionally, *Nde*I and *Xho*I restriction sites were added at the 5′ and 3′ ends of the sequence, respectively. The optimized sequence was synthesized and cloned into pUC57 vector by GenScript (Township, NJ, USA). Afterward, a *Nde*I-*Xho*I fragment containing the *orf18* gene was released from pUC57 and sub-cloned into pET21a(+) vector (EMD Biosciences, San Diego, CA, USA), which introduces a C-terminal 6 His-tag. The construct (pET21a-*dpo7*) was verified by DNA sequencing using vector-specific primers (T7 promoter: 5′-TAATACGACTCACTATAGGG-3′ and T7 terminator 5′-GCTAGTTATTGCTCAGCGG-3′, Eurogentec, Madrid, Spain) and two internal primers (5′-TCAGAAAGATTCCACGAAGG-3′ and 5′-TAATGGCCATGTGAGCATC-3′, Eurogentec, Madrid, Spain).

The plasmid pET21a-*dpo7* was electroporated in *E. coli* BL21 (DE3) pLysS (Invitrogen Corporation, Gent, Belgium) and protein expression was carried out as described previously (Obeso et al., 2008) with 1 mM of IPTG for 16 h at 16°C. Five hundred milliliter culture cells were pelleted, suspended in 10 ml lysis buffer (20 mM NaH_2_PO_4_, 500 mM NaCl, 10 mM imidazole, pH 7.4) and frozen/thawed three times at -80°C. Sonication was carried out afterward 15 × 5 s pulses with 15 s recovery on ice and centrifuged at 10,000 × *g*. The supernatant containing the protein was purified using the His GraviTrap column kit (GE Healthcare Life Sciences, Buckinghamshire, UK) following the supplier’s recommendations. Wash buffer and lysis buffer were composed of 20 mM NaH_2_PO_4_, 500 mM of NaCl, pH 7.4 with 50 or 500 mM imidazole, respectively. Protein purity was estimated by SDS-PAGE and the predicted amino acid sequence was confirmed by mass spectrometry (MALDI-TOF/TOF) as previously described (García et al., 2004). Protein amount was quantified by the Quick Start Bradford Protein Assay (BioRad, Hercules, CA, USA). Prior to the activity assays, the purified Dpo7 was dialyzed for 16 h against PBS buffer (137 mM NaCl 2.7 mM KCl 10 mM Na_2_HPO_4_ 2 mM KH_2_PO_4_; pH 7.4).

### Biofilm Assays

Biofilms of staphylococcal strains were grown into a TC Microwell 96U w/lid nunclon D SI plates (Thermo Scientific, NUNC, Madrid, Spain). Overnight cultures were diluted in TSBg [TSB supplemented with 0.25% w/v D-(+)-glucose] up to 10^6^ CFU/ml and 200 μl were poured into each well and incubated for 24 h at 37°C. Wells were washed twice with sterile phosphate-buffered saline (PBS buffer) (137 mM NaCl, 2.7 mM KCl, 10 mM Na_2_HPO_4_ and 2 mM KH_2_PO_4_; pH 7.4). After treatment of biofilms (see below), cultivable bacteria released in the supernatant were counted by plating on TSA serial dilutions. Moreover, cultivable adhered bacteria were determined by scratching twice with a sterile swab and then immersed into 9 ml of PBS buffer followed by a vigorous shaking for 1 min. Finally, several decimal dilutions were plated onto TSA and incubated at 37°C. Biomass quantification of biofilm adhered to the surface of wells was carried out as previously described (O’Neill et al., 2008). Briefly, wells were treated with crystal violet (0.1% w/v) for 15 min, followed by a gentle wash with water and de-staining in acetic acid (33% v/v). Finally, absorbance was measured at 595 nm. To determine the matrix composition, the biofilms were washed with PBS and then treated for 1 h at 37°C with a solution of 10 mM sodium metaperiodate in 50 mM sodium acetate buffer (pH 4.5) (to disrupt the extracellular polysaccharides), with 100 mg/ml of proteinase K (Sigma, Madrid, Spain) in 20 mM Tris HCl (pH 7.5) and 100 mM NaCl, or with 100 mg/ml of DNaseI (Sigma, Madrid, Spain) in 150 mM of NaCl and 1 mM CaCl_2_ (Kaplan et al., 2004; Holland et al., 2011). After treatments, the biofilms were washed with water, stained with crystal violet, and the absorbance measured as described above. All assays were performed using four biological replicates.

### Characterization of Dpo7 Activity

To determine the optimal conditions for Dpo7 activity, different concentrations of the protein (0–1.5 μM) or PBS buffer for control purposes, were added to 24 h-preformed biofilms of *S. epidermidis* F12, and incubated for different times (30 min – 24 h) at temperatures ranging from 22 to 37°C. Biofilm removal was quantified by enumeration of cultivable bacteria and crystal violet staining (see above).

The activity of the protein was also tested against the biofilms formed by other staphylococcal strains using 0.15 μM of Dpo7 for 3 h at 37°C. All experiments were performed in triplicate.

The ability of Dpo7 to prevent biofilm formation was also tested using a conventional broth microdilution technique. Two-fold dilutions of Dpo7 (0–1.5 μM) in TSBg were added to TC Microwell 96U w/lid nunclon D SI plates (Thermo Scientific, NUNC, Madrid, Spain) in order to test biofilm formation and to 96-Well Microtiter^TM^ Microplates (Thermo Scientific, NUNC, Madrid, Spain) to assess planktonic bacterial growth. Each well was inoculated with 10^6^ CFU/well of bacteria. Plates were incubated at 37°C for 24 h. Biofilm formation was quantified by crystal violet staining and measuring the absorbance at 595 nm. Planktonic growth was determined by measuring the absorbance at 600 nm of each supernatant.

Dpo7 activity against extracellular material was assessed by using exponential cultures (OD_600_ = 0.6) of *S. epidermidis* F12 in TSBg. Cells were suspended in PBS buffer containing 0.15 μM of Dpo7 and incubated for 3 h at 37°C. Three microliter of the cells diluted in 10 μl of 1% Congo red aqueous solution (Sigma–Aldrich, St. Louis, MO, USA) were spread onto a glass slide and air-dried. To visualize the extracellular material, staining with Maneval’s solution was performed as previously described (Cornelissen et al., 2011).

Quantification of lytic activity of Dpo7 was tested against live *S. epidermidis* F12 cells prepared as previously described (Becker et al., 2009), using the turbidity reduction assay (Obeso et al., 2008).

The pH stability of the protein Dpo7 was tested by dilution (1.5 μM) into the Britton–Robinson pH universal buffer (150 mM KCl, 10 mM KH_2_PO_4_, 10 mM sodium citrate, 10 mM H_3_BO_3_, adjusted within a pH 3–11 range, and subsequent maintenance at room temperature for 1 h. The protein was then diluted 10-fold in PBS buffer. For control purposes, Britton–Robinson buffer was diluted 10-fold in PBS buffer. Similarly, the temperature stability was examined after incubation of 0.15 μM of Dpo7 in PBS buffer for 30 min at different temperatures (ranging from 40 to 90°C). The activity of the protein after these treatments was tested against 24 h-biofilms of *S. epidermidis* F12 as indicated above.

### Statistical Analysis

A one-way analysis of variance (ANOVA) and the LSD test was carried out to establish any significant differences regarding the adhered cells and cells number in the supernatant and biomass between the control and the treated biofilms. The differences were expressed as the mean ± standard error and the level of significance was established at *P <* 0.05 (SPSS11.0 Software for windows; Chicago, IL, USA).

## Results

### Dpo7 is Able to Remove *S. epidermidis* Extracellular Material in Both Biofilms and Planktonic Cells

**FIGURE 1:**
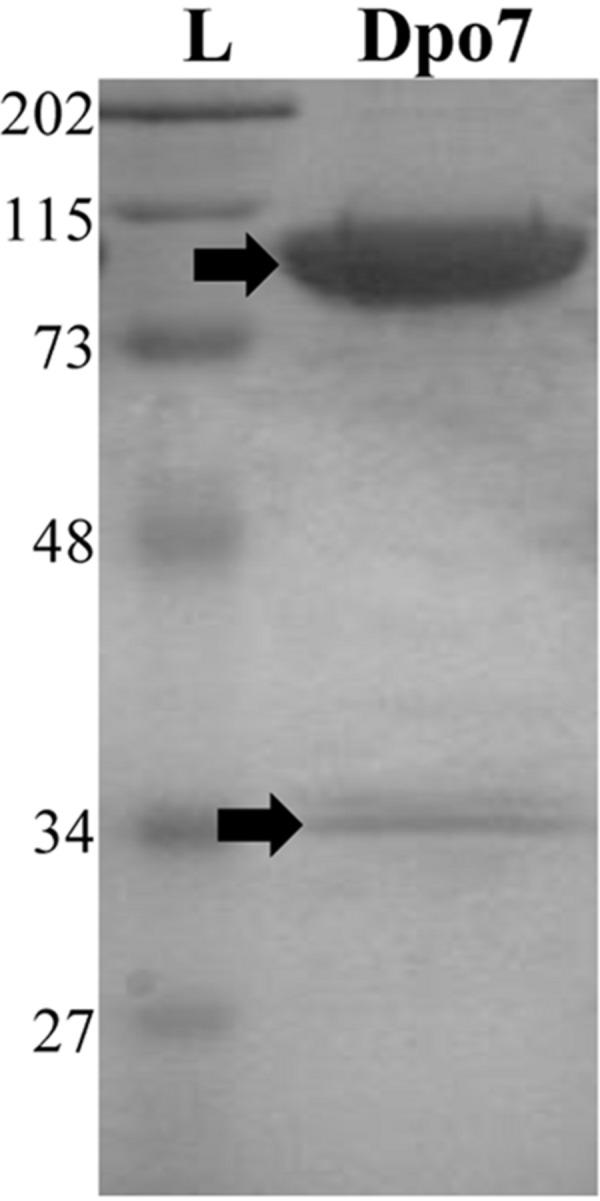
**Purification of the recombinant exopolysaccharide depolymerase Dpo7 from *Escherichia coli* BL21(DE3) pLys pET21a-*dpo7*.** L: Standard molecular weight marker in kDa (Low Range Molecular Weight SDS-PAGE Standards, BioRad); Fraction eluted from nickel affinity chromatography containing purified Dpo7. Bands indicated with a black arrow were identified by mass spectrometry.

To determine the activity of the recombinant protein Dpo7, a synthetic gene with optimized codons was used for expression in *E. coli* BL21 (DE3) pLysS as an N-terminal 6×-His-tagged fusion, allowing purification by immobilized metal chelate affinity chromatography. The purity of the protein was estimated to be 95% as assessed by SDS-PAGE analysis (**Figure 1**). A main band of about 98 kDa corresponding with the molecular weight of Dpo7 was observed. A minor additional band (32 kDa) was identified by mass-spectrometry as a degradation product of Dpo7. Stocks of about 15 μM of protein were routinely obtained in these conditions.

**FIGURE 2:**
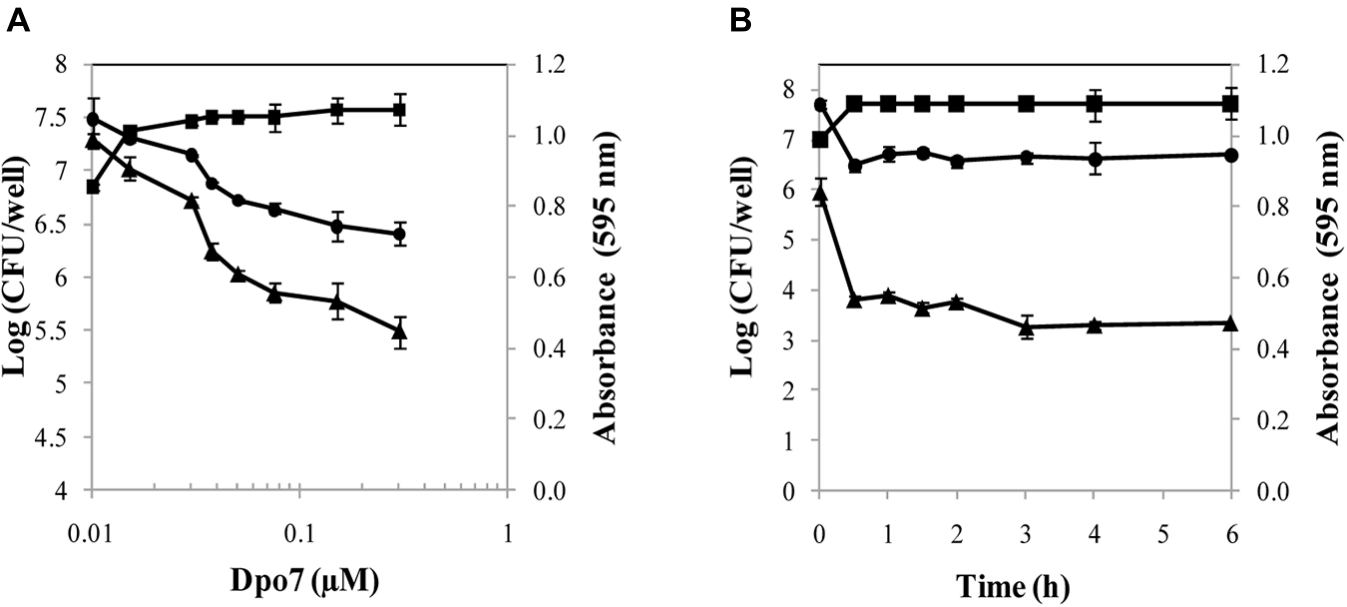
**Activity of Dpo7 against 24 h-biofilms of *Staphylococcus epidermidis* F12 in PBS buffer at 37°C.** Biofilm disruption was determined using **(A)** different concentrations of Dpo7 (μM) for 3 h at 37°C and **(B)** 0.15 μM of Dpo7 throughout time ranging 30 min to 6 h. Total attached biomass was measured by crystal violet staining after treatment and expressed as *A_*595*_* units (▲); Adhered cultivable bacteria (●) and supernatant cultivable bacteria (■) are expressed as Log (CFU/well). Each value corresponds to the mean ± standard deviation of three independent experiments.

The activity of the recombinant Dpo7 was characterized by treatment of 24 h-biofilms of the strain *S. epidermidis* F12 with different concentrations of Dpo7 for 3 h at 37°C (**Figure 2A**). Total biomass that remained attached after the Dpo7 treatment was quantified by crystal violet staining. The maximum biofilm disruption (43%) was obtained with 0.075 μM and higher concentrations (up to 1.5 μM) did not improve significantly this result (*P* < 0.05). Moreover, the number of cultivable bacteria was also determined after Dpo7 treatment. The maximum removal of attached cells (about 1 log unit; 92% of the total) was obtained treating the biofilms with 0.15 μM of the recombinant protein. This value correlates with the increase in the number of cells in the supernatant (**Figure 2A**) but the total cell number (adhered plus planktonic) remained constant (data not shown), indicating that biofilm-associated cells were released to the planktonic state without any lytic activity of Dpo7. A turbidity reduction assay confirmed the absence of lytic activity in Dpo7. There were no statistical differences between the control and the treated cells (data not shown). These results support the hypothesis that the activity of Dpo7 is related with the degradation of the extracellular material that surrounds the bacteria inside the biofilm rather than lysis of the cells.

Once the Dpo7 minimum concentration to disrupt 50% of the biofilm was established, an assay with different time exposures was carried out at 37°C (**Figure 2B**). For control purposes, the number of cultivable bacteria and biomass staining were recorded in biofilms treated only with PBS buffer for each time, but no differences were observed during the assay. The results showed that the protein was active and most of biofilm matrix degradation took place within the first 30 min. The minimum adhered cells and the maximum cells in the supernatants were also detected 30 min after treatment, remaining stable until the end of the exposure (**Figure 2B**). Longer times of incubation (until 24 h) did not change these results.

**FIGURE 3:**
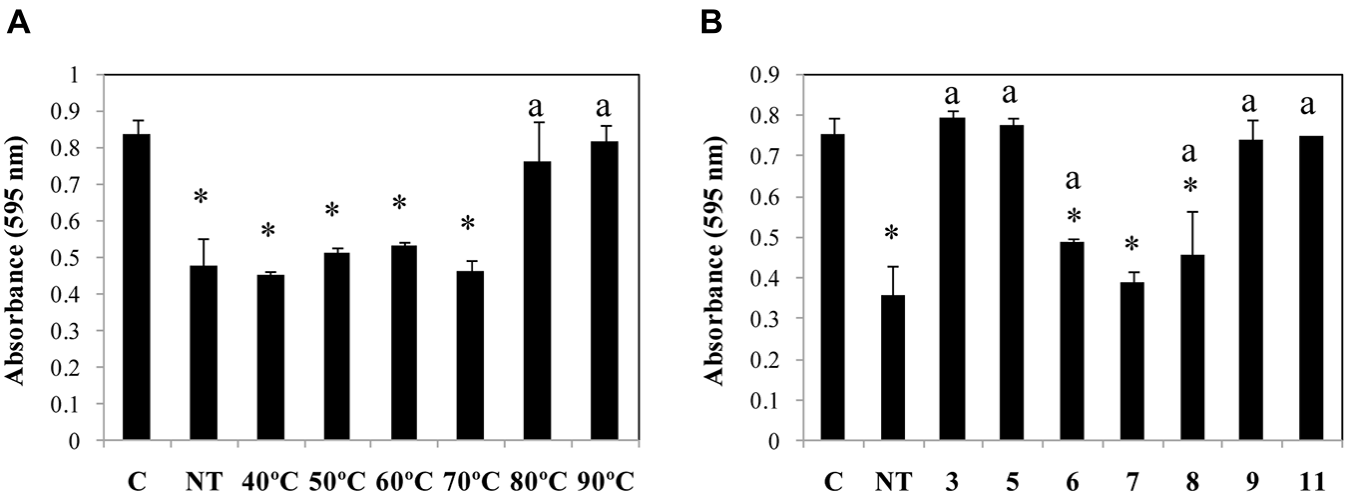
**Temperature **(A)** and pH **(B)** stability of Dpo7.** Temperature stability was tested by incubation of Dpo7 (0.15 μM) for 30 min and pH stability after maintenance at room temperature for 1 h. C: control biofilm of *S. epidermidis* F12 without Dpo7 treatment; NT: activity of Dpo7 at pH 7.4 without temperature treatment. Values represent the mean ± standard deviation of three independent experiments. Bars having an asterisk are statistically significant different from the control and bars with a lower case ‘a’ indicate a statistically significant difference between the biofilm treatment with standard Dpo7 and the activity after thermal or pH treatment (ANOVA; *P* < 0.05).

The optimum parameters for Dpo7 activity were determined by incubation at several temperatures (22, 32, and 37°C). Dpo7 was active in all temperatures tested with increasing activity at increasing temperature (25, 30, and 44% of biomass was removed at 22, 32, and 37°C, respectively). Dpo7 was found to be quite thermostable as heat treatments up to temperatures of 70°C did not significantly affect the enzymatic activity (**Figure 3A**). Dpo7 was also stable in a range of pH from 6 to 8 (**Figure 3B**). After 90 days of storage a 4°C, no decrease of activity was observed (data not shown).

**FIGURE 4:**
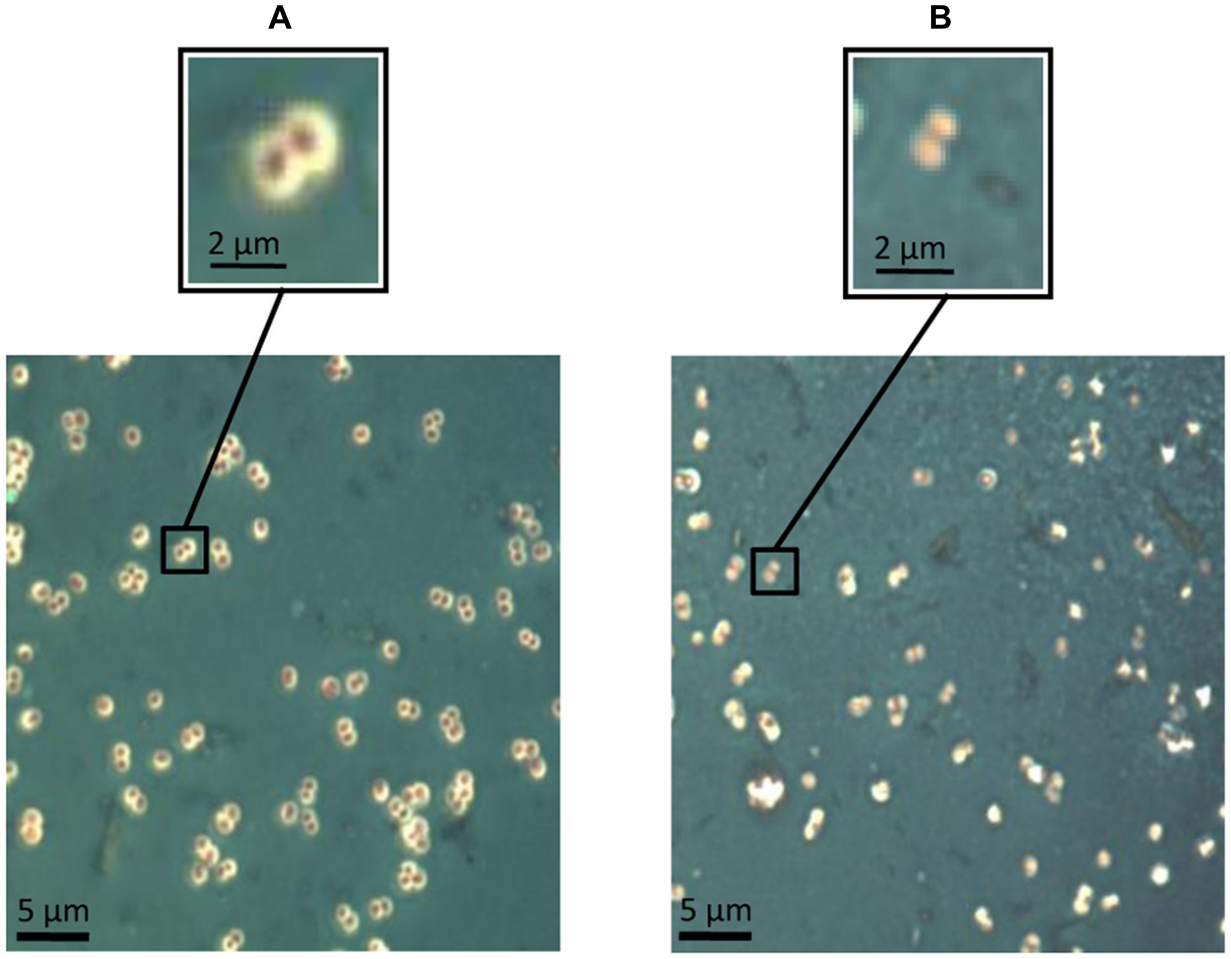
**Maneval’s staining of extracellular material in exponential-phase cultures of *S. epidermidis* F12 **(A)** before treatment and **(B)** after treatment with 0.15 μM of Dpo7 for 3 h at 37°C.** A magnification of the figure allows for the comparison of the presence/absence of polysaccharide matrix represented by a white halo surrounding the cell.

A qualitative approach about the potential of Dpo7 to degrade the extracellular material formed by *S. epidermidis* F12 in liquid cultures was obtained by staining and optical microscopy (**Figure 4**). Cells grown to exponential phase were incubated with 0.15 μM of Dpo7 for 3 h at 37°C. Microscopic analysis showed that non-treated cells are closely associated into small clusters surrounded by a thin layer of negatively stained capsular material (**Figure 4A**). After treatment with exopolysaccharide (EPS) depolymerase Dpo7, the capsule is generally thinner or drastically disrupted, and remaining capsular material devoid of bacteria can be observed (**Figure 4B**).

### The Exopolysaccharide is the Main Substrate of Dpo7

**FIGURE 5:**
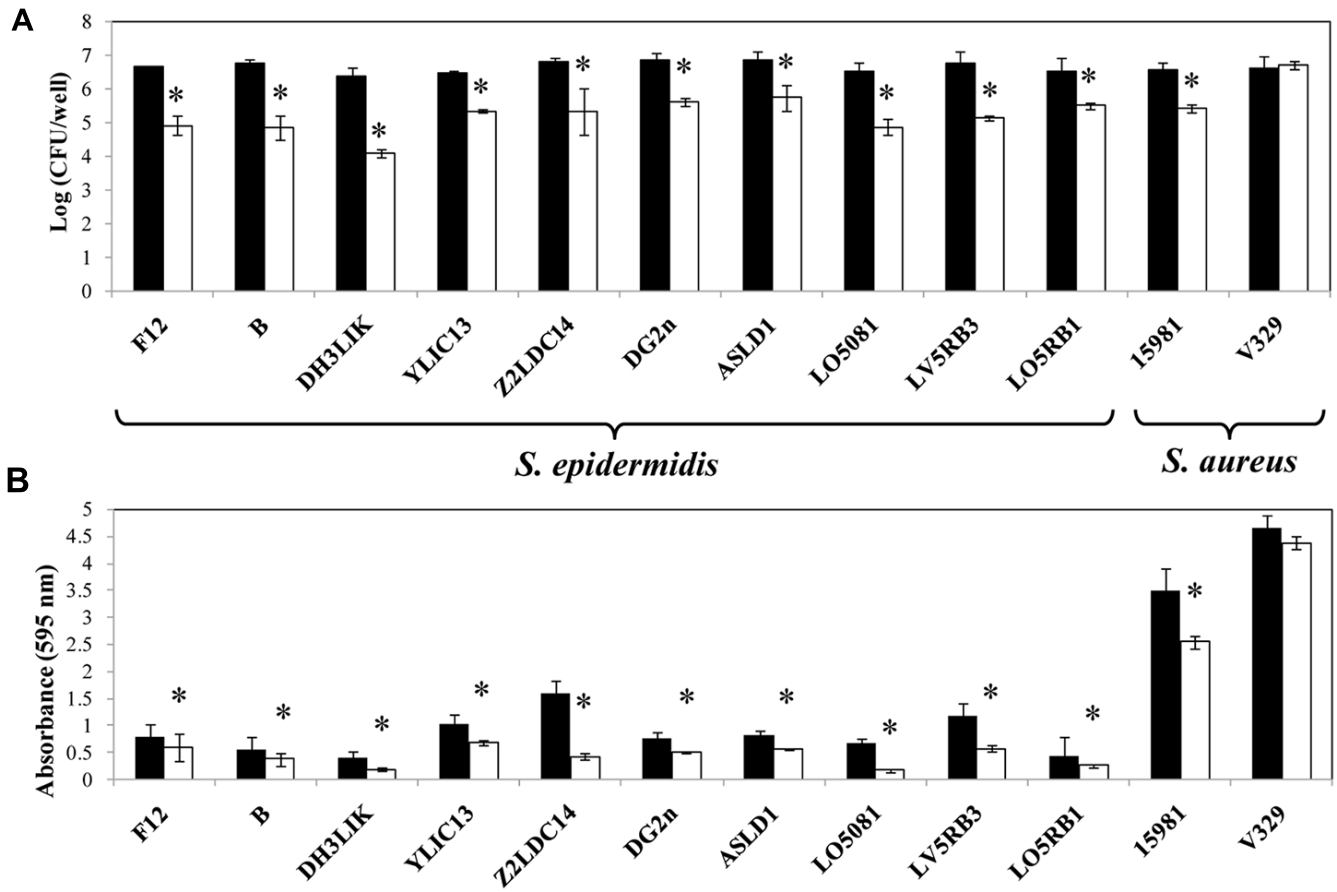
**Removal of 24 h *S. epidermidis* and *S. aureus* biofilms after addition of Dpo7 (0.15 μM) for 3 h at 37°C. (A)** Adhered cultivable bacteria and **(B)** crystal violet staining of control biofilms (black) and treated biofilms (white). Means and standard deviations were calculated from three biological replicates. Bars having an asterisk are significantly different from the control (ANOVA; *P* < 0.05).

To determine the specificity of Dpo7 for the components of *S. epidermidis* biofilms, we tested its activity against biofilms with different biochemical matrix compositions. Our *S. epidermidis* and *S. aureus* strains collection was tested for matrix composition by using treatment with either NaIO_4_, proteinase K and DNaseI, specific for exopolysaccharidic, proteinaceous and DNA matrix, respectively. An estimation of the matrix composition was made as function of the percentage of biofilm removal after treatments. The results showed that all *S. epidermidis* strains tested produced a matrix composed mainly of EPS as the highest percentage of biofilm removal was obtained after treatment with NaIO_4_. Strains *S. aureus* 15981 and *S. aureus* V329 formed EPS and proteinaceous biofilms, respectively (**Table 2**). Dpo7 assays against 24 h-biofilms of *S. epidermidis* strains showed a significant reduction in adhered cells from biofilms containing an EPS matrix (**Figure 5A**). The decrease ranged from 1 log-unit in the biofilm formed by *S. epidermidis* LO5RB1 to 2.3 log-units in *S. epidermidis* DH3LIK biofilm. Moreover, an increase in the number of cells was observed in the supernatant (data not shown). As expected, the number of adhered cells in *S. aureus* V329 biofilms was not reduced due to its proteinaceous biofilm matrix but a clear reduction was observed in *S. aureus* 15981 (**Figure 5A**). These results were confirmed by staining the total biomass with crystal violet. Dpo7 activity against EPS biofilms showed a reduction of biomass ranging from 31% in *S. epidermidis* ASLD1 to 75% in *S. epidermidis* LO5081, remaining unaffected in *S. aureus* V329 (**Figure 5B**).

**Table 2:** Estimation of the extracellular components in biofilms formed by staphylococcal strains through the percentage of biofilm removing after treatment.

	Strain	% of removed biofilm		
		NaIO_4_	Proteinase K	DNase I
*S. epidermidis*	F12	77.7 ± 9.0	1.9 ± 0.3	14 ± 5.9
	B^∗^	76.2 ± 8.2	2.7 ± 1.4	14.9 ± 3.1
	DH3LIK	85 ± 3.8	5.9 ± 1.9	6.2 ± 1.2
	YLIC13	71.8 ± 1.3	8 ± 1.8	22 ± 2.4
	Z2LDC14	73.4 ± 2.8	7.7 ± 2.7	32.3 ± 5.5
	DG2n^∗^	75.2 ± 4.6	3.9 ± 1.5	24.7 ± 10.4
	ASLD1	73.3 ± 5.6	0.5 ± 0.3	24.2 ± 7.1
	LO5081	81.4 ± 9.6	9.7 ± 3.3	36.6 ± 3.3
	LV5RB3	76.7 ± 4.2	8.1 ± 7.7	23.1 ± 6.4
	LO5RB1	86.2 ± 6.0	6.1 ± 2.7	23.1 ± 9.5
*S. aureus*	15981^∗^	88.7 ± 2.5	3.5 ± 0.9	1.6 ± 0.8
	V329^∗^	3.2 ± 5.1	88.7 ± 0.1	49.4 ± 2.2

*^∗^Results published in Gutiérrez et al. (2014).*

### Dpo7 Prevents the Biofilm Formation on Polystyrene Surfaces

**FIGURE 6:**
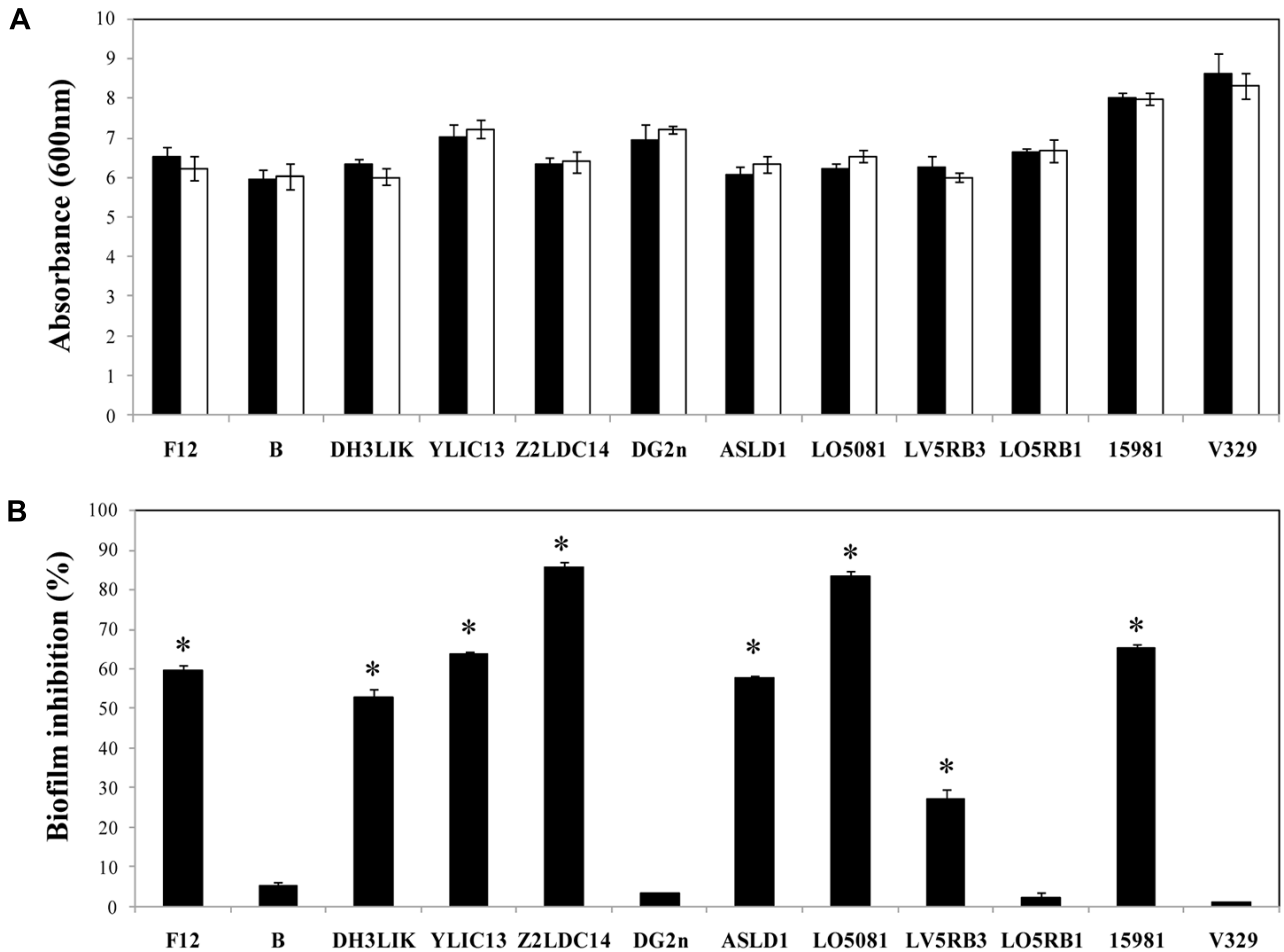
**Biofilm growth inhibition in presence of Dpo7 (0.15 μM). (A)** Planktonic growth of the cultures was determined by absorbance measurement at 600 nm after 24 h of incubation at 37°C. **(B)** Percentage of biofilm inhibition was calculated by crystal violet staining of adhered cells after growth of 24 h biofilms. Black bars represent the control values and white bars represent the values of wells treated with Dpo7. Means and standard deviations were calculated from three biological replicates. Bars having an asterisk are significantly different from the control (ANOVA; *P* < 0.05).

In addition to the evaluation of Dpo7 to prevent biofilm formation in staphylococcal strains, we tested the effect of the protein on the planktonic growth of the strains. Microwell plates filled with TSBg supplemented with Dpo7 (0–1.5 μM) were inoculated with staphylococcal strains and the growth at 37°C was monitored by DO_600_ after 24 h of incubation. No statistically significant differences were observed between planktonic cultures treated and non-treated with Dpo7 at any assayed concentration. **Figure 6A** shows results obtained at 0.15 μM Dpo 7 but higher concentrations (up to 1.5 μM) did not affect planktonic growth.

In order to assess the ability of Dpo7 to prevent biofilm formation, staphylococcal strains were also grown in the presence of different concentrations (0–1.5 μM) of Dpo7 and checked for biofilm formation by crystal violet staining. The presence of 0.15 μM of Dpo7 inhibited biofilm formation as total biomass adhered to the polystyrene surface decreased from 27% in *S. epidermidis* LV5RB3 to 85% in *S. epidermidis* Z2LDC14 (**Figure 6B**). Higher concentrations of Dpo7 (up to 1.5 μM) did not improve these results (data not shown). However, the presence of the Dpo7 did not affect biofilm formation in the case of *S. epidermidis* B, DG2n, LO5RB1 and *S. aureus* V329 (**Figure 6B**).

## Discussion

In this study, we have purified a phage-derived EPS depolymerase, Dpo7, which degrades the EPS matrix of *S. epidermidis* and *S. aureus* biofilms. Apart from Dispersin B, an *N*-acetylglucosaminidase enzyme produced by *Aggregatibacter actinomycetemcomitans* (Izano et al., 2008), no other enzymes degrading staphylococcal biofilm matrix formed by polysaccharides have been described so far. This finding prompted us to study Dpo7 as a strategy for controlling the colonization and biofilm formation by staphylococcal pathogens. The presence of depolymerases is common among phages, playing a putative role in phage adsorption and infection (Cornelissen et al., 2011, 2012). Similar proteins to Dpo7 are also present in three other *Siphoviridae* phages infecting *S. epidermidis* (Daniel et al., 2007; Gutiérrez et al., 2012). All phage EPS depolymerases characterized to date are enzymes that hydrolyze polysaccharides or polysaccharide derivatives and are common constituents of the tail spikes (Yan et al., 2014). For instance, phage EPS depolymerases include endorhamnosidases (Muller et al., 2008; Andres et al., 2010), alginate lyases (Glonti et al., 2010), endosialidases (Jakobsson et al., 2007) and hyaluronidases (Smith et al., 2005). Regarding Dpo7, it is reasonable to hypothesize that it is associated to the capsid. Indeed, Dpo7 amino acid sequence showed 99% homology with a pre-neck appendage protein encoded by *S. epidermidis* bacteriophage CNPH82 and high similarity in the tridimensional structure with phi29 gp12 neck protein (Gutiérrez et al., 2012).

The protein Dpo7 contains a predicted pectin lyase domain, the structure of which is composed of a right-handed β-helix (Gutiérrez et al., 2012). Proteins containing these repeats are usually enzymes with polysaccharide substrates, and this topology is shared by several proteins, including bacterial pectate lyases, fungal and bacterial galacturonases. Also, the phage tail spike protein HylP1, encoded by l-like phage infecting *Streptococcus pyogenes*, is a hyaluronidase with a similar structure (Smith et al., 2005). Our results show that Dpo7 is involved in the degradation of the EPS biofilm matrix of staphylococcal strains. The ability of Dpo7 to disperse polysaccharide staphylococcal preformed biofilms, but not the proteinaceous biofilm formed by the strain *S. aureus* V329 supports this hypothesis. Degradation of proteins associated with *S. aureus* biofilm formation and host–pathogen interaction was previously described by *S. epidermidis* protease Esp (Sugimoto et al., 2013).

In spite of the low identity (13%) and similarity (21%) percentages between the amino acid sequences of Dpo7 and DispersinB^®^, the enzymatic activity and the optimum conditions of Dpo7 were similar to those of DispersinB^®^. Of note, the last one has been proved to be effective removing over 85% of the biomass of some staphylococcal biofilms when applying 40 or 50 μg ml^-1^ (Kaplan et al., 2003; Izano et al., 2008; Turk et al., 2013). The activity of Dpo7 was not dependent on the ability of the strains to form weaker or stronger biofilms but on the nature of the matrix. Indeed, the highest percentage of biofilm removal was obtained against *S. epidermidis* Z2LDC14 and *S. epidermidis* LO5081, which showed a high and a moderate ability to form biofilm, respectively, from among *S. epidermidis* strains. We also observed that concentrations over 0.15 μM did not improve biofilm dispersion even if time of treatment was increased. The maintenance of the biofilm after the treatment could be due to the heterogeneity of the biofilm matrix or to a low diffusion of the protein within the biofilm. In EPS biofilms, although the majority of the extracellular matrix is composed of polysaccharides, interactions with eDNA and proteins should not be discarded (Izano et al., 2008) therefore limiting the potential of Dpo7 to remove the biofilm completely. This limitation was also observed when using DispersinB^®^ to remove biofilms formed by *S. pseudintermedius* (Turk et al., 2013). The activity of this enzyme was dependent on the proportion of the constituents of the biofilm matrix, which varies with the growth conditions and among the different strains (Chaignon et al., 2007). Moreover, PIA/PNAG molecules do not have a definite structure and are not well-defined substrates, varying in length and charge (Mack et al., 1996). It has been demonstrated that the chain length of PIA/PNAG increased the catalytic efficiency of DispersinB^®^ (Fazekas et al., 2012). Similarly, Dpo7 may control EPS staphylococcal biofilms, but its activity could be limited by the nature of the biofilm matrix, requiring the use of combined treatments to completely eradicate the biofilm. Combined treatments of DispersinB^®^ with antibacterials such as triclosan or cefamandole nafate reduced colonization and biofilm formation by *S. aureus, S. epidermidis*, and *E. coli* in coated medical devices (Donelli et al., 2007; Darouiche et al., 2009). Moreover, the development of an engineered enzymatic bacteriophage (T7) modified to express DispersinB^®^ during infection, enhanced the ability of the phage to reduce biofilms formed by *E. coli* (Lu and Collins, 2007).

The relatively low activity of Dpo7 against extracellular material allows us to speculate about the role of this protein in the viral particle. Dpo7 might be implicated in the access of phage to the bacterial surface without a total disaggregation of the biofilm structure. Moreover, cells susceptible to phage attack should be located in the outer zone of biofilms, where nutrients and oxygen availability allow them to grow actively. Therefore, the biological function of Dpo7 might not be the highly effective degradation of extracellular EPS matrix but to open a local trajectory to enable virion/cell contact.

A major finding of this study was that treatment of polystyrene surfaces with Dpo7 inhibits the colonization by staphylococcal strains. It seems that removal of the extracellular material at the early stages of the culture is sufficient to prevent the adhesion of most strains to the polystyrene surfaces. As deduced from our results, Dpo7 is also able to remove extracellular material from planktonic cells, resulting in the inhibition of biofilm formation. This is in accordance with previous results obtained adding DispersinB^®^ for biofilm prevention of several bacteria specific strains such as *S. epidermidis, E. coli*, or *Yersinia pestis*, which produce polysaccharidic matrixes (Itoh et al., 2005), and it was also useful to reduce but not completely inhibit biofilm formation of several strains of *S. pseudintermedius* (Turk et al., 2013).

All these results in addition to the lack of an antibacterial effect of Dpo7 indicates that this enzyme would be used to remove the extracellular material in staphylococcal strains to reduce their virulence, since it has been shown that *S. epidermidis* mutants deficient in the ability to synthesize PIA/PNAG are avirulent in animal models of infection (Shiro et al., 1994). Similarly, biofilm production by *S. aureus* is important for high-level virulence in murine models of systemic infection, likely due to protection of cells from host defenses (Kropec et al., 2005). Thus, Dpo7 may also be considered as an antivirulence compound. Generally, resistance development against antivirulence compounds is expected to be lower than against antibacterial compounds, as the bacteria are only disarmed and not killed (Allen et al., 2014). The efficacy of anti-biofilm enzymes has been proven with DispersinB^®^ for the treatment of infectious diseases in a chronic wound mouse model of MRSA infection (Gawande et al., 2014), and in the reduction of colonization of catheters by *S. aureus* (Darouiche et al., 2009). The removal of capsular polysaccharide by the phage-encoded enzyme endosialidase E reduces the virulence of *E. coli* K1 in a non-invasive model in neonatal rats (Mushtaq et al., 2005). Similarly, depolymerase enzymes encoded by phages infecting *Erwinia amylovora* have been proposed in the control of plant diseases (Kim et al., 2004). In addition, the removal of polymeric extracellular material might improve the action of antibiotics against bacteria forming biofilms, since physical interference could be avoided (Farber et al., 1990; Alkawash et al., 2006).

This study supports that phages possess structural components such as the novel EPS depolymerase (Dpo7) that inhibit biofilm formation and disperses preformed biofilms of *S. epidermidis* and *S. aureus*. These kinds of proteins might constitute a new approach to remove biofilms, thus improving the treatment of biofilm-associated recalcitrant infections.

## Author Contributions

DG, YB, LR-R, BM, AR, RL, and PG conceived and designed the experiments. DG performed the experiments. DG, YB, AR, PG, and RL analyzed the data. DG, YB, LR, BM, AR, RL, and PG wrote the paper.

## Conflict of Interest Statement

The authors declare that the research was conducted in the absence of any commercial or financial relationships that could be construed as a potential conflict of interest.

## References

[B1] Alkawash M. A. Soothill J. S. Schiller N. L. (2006). Alginate lyase enhances antibiotic killing of mucoid *Pseudomonas aeruginosa* in biofilms. *APMIS* 114 131–138. 10.1111/j.1600-0463.2006.apm_356.x 16519750

[B2] Allen R. C. Popat R. Diggle S. P. Brown S. P. (2014). Targeting virulence: can we make evolution-proof drugs? *Nat. Rev. Microbiol.* 12 300–308. 10.1038/nrmicro3232 24625893

[B3] Andres D. Hanke C. Baxa U. Seul A. Barbirz S. Seckler R. (2010). Tailspike interactions with lipopolysaccharide effect DNA ejection from phage P22 particles in vitro. *J. Biol. Chem.* 285 36768–36775. 10.1074/jbc.M110.169003 20817910 PMC2978605

[B4] Becker S. C. Dong S. Baker J. R. Foster-Frey J. Pritchard D. G. Donovan D. M. (2009). LysK CHAP endopeptidase domain is required for lysis of live staphylococcal cells. *FEMS Microbiol. Lett.* 294 52–60. 10.1111/j.1574-6968.2009.01541.x 19493008

[B5] Cerca N. Oliveira R. Azeredo J. (2007). Susceptibility of *Staphylococcus epidermidis* planktonic cells and biofilms to the lytic action of *Staphylococcus* bacteriophage K. *Lett. Appl. Microbiol.* 45 313–317. 10.1111/j.1472-765X.2007.02190.x 17718845

[B6] Chaignon P. Sadovskaya I. Ragunah C. Ramasubbu N. Kaplan J. B. Jabbouri S. (2007). Susceptibility of staphylococcal biofilms to enzymatic treatments depends on their chemical composition. *Appl. Microbiol. Biotechnol.* 75 125–132. 10.1007/s00253-006-0790-y 17221196

[B7] Christner M. Franke G. C. Schommer N. N. Wendt U. Wegert K. Pehle P. (2010). The giant extracellular matrix-binding protein of *Staphylococcus epidermidis* mediates biofilm accumulation and attachment to fibronectin. *Mol. Microbiol.* 75 187–207. 10.1111/j.1365-2958.2009.06981.x 19943904

[B8] Cornelissen A. Ceyssens P. J. Krylov V. N. Noben J. P. Volckaert G. Lavigne R. (2012). Identification of EPS-degrading activity within the tail spikes of the novel *Pseudomonas putida* phage AF. *Virology* 434 251–256. 10.1016/j.virol.2012.09.030 23084421

[B9] Cornelissen A. Ceyssens P. J. T’Syen J. Van Praet H. Noben J. P. Shaburova O. V. (2011). The T7-related *Pseudomonas putida* phage phi15 displays virion-associated biofilm degradation properties. *PLoS ONE* 6:e18597 10.1371/journal.pone.0018597 PMC3079711 21526174

[B10] Cucarella C. Solano C. Valle J. Amorena B. Lasa I. Penadés J. R. (2001). Bap, a *Staphylococcus aureus* surface protein involved in biofilm formation. *J. Bacteriol.* 183 2888–2896. 10.1128/JB.183.9.2888-2896.2001 11292810 PMC99507

[B11] Daniel A. Bonnen P. E. Fischetti V. A. (2007). First complete genome sequence of two *Staphylococcus epidermidis* bacteriophages. *J. Bacteriol.* 189 2086–2100. 10.1128/JB.01637-06 17172342 PMC1855768

[B12] Darouiche R. O. Mansouri M. D. Gawande P. V. Madhyastha S. (2009). Antimicrobial and antibiofilm efficacy of triclosan and DispersinB combination. *J. Antimicrob. Chemother.* 64 88–93. 10.1093/jac/dkp158 19447791

[B13] David M. Z. Daum R. S. (2010). Community-associated methicillin-resistant *Staphylococcus aureus*: epidemiology and clinical consequences of an emerging epidemic. *Clin. Microbiol. Rev.* 23 616–687. 10.1128/CMR.00081-09 20610826 PMC2901661

[B14] Delgado S. Arroyo R. Jiménez E. Maín M. L. del Campo R. Fernández L. (2009). *Staphylococcus epidermidis* strains isolated from breast milk of women suffering infectious mastitis: potential virulence traits and resistance to antibiotics. *BMC Microbiol.* 9:82 10.1186/1471-2180-9-82 PMC2685400 19422689

[B15] Domenech M. García E. Moscoso M. (2011). In vitro destruction of *Streptococcus pneumoniae* biofilms with bacterial and phage peptidoglycan hydrolases. *Antimicrob. Agents Chemother.* 55 4144–4148. 10.1128/AAC.00492-11 21746941 PMC3165314

[B16] Donelli G. Francolini I. Romoli D. Guaglianone E. Piozzi A. Ragunath C. (2007). Synergistic activity of dispersin B and cefamandole nafate in inhibition of staphylococcal biofilm growth on polyurethanes. *Antimicrob. Agents Chemother.* 51 2733–2740. 10.1128/AAC.01249-06 17548491 PMC1932551

[B17] Farber B. F. Kaplan M. H. Clogston A. G. (1990). *Staphylococcus epidermidis* extracted slime inhibits the antimicrobial action of glycopeptide antibiotics. *J. Infect. Dis.* 161 37–40. 10.1093/infdis/161.1.37 2295856

[B18] Fazekas E. Kandra L. Gyemant G. (2012). Model for beta-1,6-N-acetylglucosamine oligomer hydrolysis catalysed by DispersinB, a biofilm degrading enzyme. *Carbohydr. Res.* 363 7–13. 10.1016/j.carres.2012.09.016 23103508

[B19] García P. Rodriguez I. Suárez J. E. (2004). A -1 ribosomal frameshift in the transcript that encodes the major head protein of bacteriophage A2 mediates biosynthesis of a second essential component of the capsid. *J. Bacteriol.* 186 1714–1719. 10.1128/JB.186.6.1714-1719.2004 14996802 PMC355979

[B20] Gawande P. V. Leung K. P. Madhyastha S. (2014). Antibiofilm and antimicrobial efficacy of DispersinB(R)-KSL-W peptide-based wound gel against chronic wound infection associated bacteria. *Curr. Microbiol.* 68 635–641. 10.1007/s00284-014-0519-6 24445333

[B21] Glonti T. Chanishvili N. Taylor P. W. (2010). Bacteriophage-derived enzyme that depolymerizes the alginic acid capsule associated with cystic fibrosis isolates of *Pseudomonas aeruginosa*. *J. Appl. Microbiol.* 108 695–702. 10.1111/j.1365-2672.2009.04469.x 19709344

[B22] Gutiérrez D. Martínez B. Rodríguez A. García P. (2010). Isolation and characterization of bacteriophages infecting *Staphylococcus epidermidis*. *Curr. Microbiol.* 61 601–608. 10.1007/s00284-010-9659-5 20449591

[B23] Gutiérrez D. Martínez B. Rodríguez A. García P. (2012). Genomic characterization of two *Staphylococcus epidermidis* bacteriophages with anti-biofilm potential. *BMC Genomics* 13:228 10.1186/1471-2164-13-228 PMC3505474 22681775

[B24] Gutiérrez D. Ruas-Madiedo P. Martínez B. Rodríguez A. García P. (2014). Effective removal of staphylococcal biofilms by the endolysin LysH5. *PLoS ONE* 9:e107307 10.1371/journal.pone.0107307 PMC4159335 25203125

[B25] Gutiérrez D. Vandenheuvel D. Martínez B. Rodríguez A. Lavigne R. García P. (2015). Two phages, phiIPLA-RODI and phiIPLA-C1C, lyse mono- and dual-species *Staphylococcal* biofilms. *Appl. Environ. Microbiol.* 81 3336–3348. 10.1128/AEM.03560-14 25746992 PMC4407228

[B26] Hall-Stoodley L. Stoodley P. (2009). Evolving concepts in biofilm infections. *Cell Microbiol.* 11 1034–1043. 10.1111/j.1462-5822.2009.01323.x 19374653

[B27] Holland L. M. Conlon B. O’gara J. P. (2011). Mutation of tagO reveals an essential role for wall teichoic acids in *Staphylococcus epidermidis* biofilm development. *Microbiology* 157 408–418. 10.1099/mic.0.042234-0 21051486

[B28] Hussain M. Heilmann C. Peters G. Herrmann M. (2001). Teichoic acid enhances adhesion of *Staphylococcus epidermidis* to immobilized fibronectin. *Microb. Pathog.* 31 261–270. 10.1006/mpat.2001.0469 11747374

[B29] Hussain M. Herrmann M. von Eiff C. Perdreau-Remington F. Peters G. (1997). A 140-kilodalton extracellular protein is essential for the accumulation of *Staphylococcus epidermidis* strains on surfaces. *Infect. Immun.* 65 519–524. 9009307 10.1128/iai.65.2.519-524.1997 PMC176090

[B30] Iorio N. L. Caboclo R. F. Azevedo M. B. Barcellos A. G. Neves F. P. Domingues R. M. (2012). Characteristics related to antimicrobial resistance and biofilm formation of widespread methicillin-resistant *Staphylococcus epidermidis* ST2 and ST23 lineages in Rio de Janeiro hospitals, Brazil. *Diagn. Microbiol. Infect. Dis.* 72 32–40. 10.1016/j.diagmicrobio.2011.09.017 22100013

[B31] Itoh Y. Wang X. Hinnebusch B. J. Preston J. F. III Romeo T. (2005). Depolymerization of beta-1,6-N-acetyl-D-glucosamine disrupts the integrity of diverse bacterial biofilms. *J. Bacteriol.* 187 382–387. 10.1128/JB.187.1.382-387.2005 15601723 PMC538831

[B32] Izano E. A. Amarante M. A. Kher W. B. Kaplan J. B. (2008). Differential roles of poly-N-acetylglucosamine surface polysaccharide and extracellular DNA in *Staphylococcus aureus* and *Staphylococcus epidermidis* biofilms. *Appl. Environ. Microbiol.* 74 470–476. 10.1128/AEM.02073-07 18039822 PMC2223269

[B33] Jakobsson E. Jokilammi A. Aalto J. Ollikka P. Lehtonen J. V. Hirvonen H. (2007). Identification of amino acid residues at the active site of endosialidase that dissociate the polysialic acid binding and cleaving activities in *Escherichia coli* K1 bacteriophages. *Biochem. J.* 405 465–472. 10.1042/BJ20070177 17394421 PMC2267309

[B34] Kaplan J. B. Ragunath C. Ramasubbu N. Fine D. H. (2003). Detachment of *Actinobacillus actinomycetemcomitans* biofilm cells by an endogenous beta-hexosaminidase activity. *J. Bacteriol.* 185 4693–4698. 10.1128/JB.185.4.1399-1404.2003 12896987 PMC166467

[B35] Kaplan J. B. Ragunath C. Velliyagounder K. Fine D. H. Ramasubbu N. (2004). Enzymatic detachment of *Staphylococcus epidermidis* biofilms. *Antimicrob. Agents Chemother.* 48 2633–2636. 10.1128/AAC.48.7.2633-2636.2004 15215120 PMC434209

[B36] Kim W. S. Salm H. Geider K. (2004). Expression of bacteriophage phiEa1h lysozyme in *Escherichia coli* and its activity in growth inhibition of *Erwinia amylovora*. *Microbiology* 150 2707–2714. 10.1099/mic.0.27224-0 15289567

[B37] Kropec A. Maira-Litran T. Jefferson K. K. Grout M. Cramton S. E. Gotz F. (2005). Poly-N-acetylglucosamine production in *Staphylococcus aureus* is essential for virulence in murine models of systemic infection. *Infect. Immun.* 73 6868–6876. 10.1128/IAI.73.10.6868-6876.2005 16177366 PMC1230935

[B38] Lowy F. D. (1998). *Staphylococcus aureus* infections. *N. Engl. J. Med.* 339 520–532. 10.1056/NEJM199808203390806 9709046

[B39] Lu T. K. Collins J. J. (2007). Dispersing biofilms with engineered enzymatic bacteriophage. *Proc. Natl. Acad. Sci. U.S.A.* 104 11197–11202. 10.1073/pnas.0704624104 17592147 PMC1899193

[B40] Ma Y. Chen M. Jones J. E. Ritts A. C. Yu Q. Sun H. (2012). Inhibition of *Staphylococcus epidermidis* biofilm by trimethylsilane plasma coating. *Antimicrob. Agents Chemother.* 56 5923–5937. 10.1128/AAC.01739-12 22964248 PMC3486604

[B41] Mack D. Fischer W. Krokotsch A. Leopold K. Hartmann R. Egge H. (1996). The intercellular adhesin involved in biofilm accumulation of *Staphylococcus epidermidis* is a linear beta-1,6-linked glucosaminoglycan: purification and structural analysis. *J. Bacteriol.* 178 175–183. 8550413 10.1128/jb.178.1.175-183.1996 PMC177636

[B42] Mann E. E. Rice K. C. Boles B. R. Endres J. L. Ranjit D. Chandramohan L. (2009). Modulation of eDNA release and degradation affects *Staphylococcus aureus* biofilm maturation. *PLoS ONE* 4:e5822 10.1371/journal.pone.0005822 PMC2688759 19513119

[B43] Muller J. J. Barbirz S. Heinle K. Freiberg A. Seckler R. Heinemann U. (2008). An intersubunit active site between supercoiled parallel beta helices in the trimeric tailspike endorhamnosidase of *Shigella flexneri* Phage Sf6. *Structure* 16 766–775. 10.1016/j.str.2008.01.019 18462681

[B44] Mushtaq N. Redpath M. B. Luzio J. P. Taylor P. W. (2005). Treatment of experimental *Escherichia coli* infection with recombinant bacteriophage-derived capsule depolymerase. *J. Antimicrob Chemother.* 56 160–165. 10.1093/jac/dki177 15914489

[B45] Nostro A. Scaffaro R. D’Arrigo M. Botta L. Filocamo A. Marino A. (2013). Development and characterization of essential oil component-based polymer films: a potential approach to reduce bacterial biofilm. *Appl. Microbiol. Biotechnol.* 97 9515–9523. 10.1007/s00253-013-5196-z 23989976

[B46] Nostro A. Scaffaro R. Ginestra G. D’Arrigo M. Botta L. Marino A. (2010). Control of biofilm formation by poly-ethylene-co-vinyl acetate films incorporating nisin. *Appl. Microbiol. Biotechnol.* 87 729–737. 10.1007/s00253-010-2598-z 20414650

[B47] Obeso J. M. Martínez B. Rodríguez A. García P. (2008). Lytic activity of the recombinant staphylococcal bacteriophage PhiH5 endolysin active against *Staphylococcus aureus* in milk. *Int. J. Food Microbiol.* 128 212–218. 10.1016/j.ijfoodmicro.2008.08.010 18809219

[B48] O’Neill E. Pozzi C. Houston P. Humphreys H. Robinson D. A. Loughman A. (2008). A novel *Staphylococcus aureus* biofilm phenotype mediated by the fibronectin-binding proteins, FnBPA and FnBPB. *J. Bacteriol.* 190 3835–3850. 10.1128/JB.00167-08 18375547 PMC2395027

[B49] Otto M. (2013). Coagulase-negative staphylococci as reservoirs of genes facilitating MRSA infection: *Staphylococcal* commensal species such as *Staphylococcus epidermidis* are being recognized as important sources of genes promoting MRSA colonization and virulence. *Bioessays* 35 4–11. 10.1002/bies.201200112 23165978 PMC3755491

[B50] Patti J. M. Allen B. L. McGavin M. J. Hook M. (1994). MSCRAMM-mediated adherence of microorganisms to host tissues. *Annu. Rev. Microbiol.* 48 585–617. 10.1146/annurev.mi.48.100194.003101 7826020

[B51] Price L. B. Stegger M. Hasman H. Aziz M. Larsen J. Andersen P. S. (2012). *Staphylococcus aureus* CC398: host adaptation and emergence of methicillin resistance in livestock. *MBio* 3:e00520-12. 10.1128/mBio.00305-11 PMC3280451 22354957

[B52] Rogers K. L. Fey P. D. Rupp M. E. (2009). Coagulase-negative staphylococcal infections. *Infect. Dis. Clin. North Am.* 23 73–98. 10.1016/j.idc.2008.10.001 19135917

[B53] Rupp M. E. (2014). Clinical characteristics of infections in humans due to *Staphylococcus epidermidis*. *Methods Mol. Biol.* 1106 1–16. 10.1007/978-1-62703-736-5_1 24222451

[B54] Saising J. Dube L. Ziebandt A. K. Voravuthikunchai S. P. Nega M. Gotz F. (2012). Activity of gallidermin on *Staphylococcus aureus* and *Staphylococcus epidermidis* biofilms. *Antimicrob. Agents Chemother.* 56 5804–5810. 10.1128/AAC.01296-12 22926575 PMC3486563

[B55] Shen Y. Koller T. Kreikemeyer B. Nelson D. C. (2013). Rapid degradation of *Streptococcus pyogenes* biofilms by PlyC, a bacteriophage-encoded endolysin. *J. Antimicrob. Chemother.* 68 1818–1824. 10.1093/jac/dkt104 23557924

[B56] Shiro H. Muller E. Gutiérrez N. Boisot S. Grout M. Tosteson T. D. (1994). Transposon mutants of *Staphylococcus epidermidis* deficient in elaboration of capsular polysaccharide/adhesin and slime are avirulent in a rabbit model of endocarditis. *J. Infect. Dis.* 169 1042–1049. 10.1093/infdis/169.5.1042 8169389

[B57] Smith N. L. Taylor E. J. Lindsay A. M. Charnock S. J. Turkenburg J. P. Dodson E. J. (2005). Structure of a group A streptococcal phage-encoded virulence factor reveals a catalytically active triple-stranded beta-helix. *Proc. Natl. Acad. Sci. U.S.A.* 102 17652–17657. 10.1073/pnas.0504782102 16314578 PMC1308890

[B58] Sugimoto S. Iwamoto T. Takada K. Okuda K. Tajima A. Iwase T. (2013). *Staphylococcus epidermidis* Esp degrades specific proteins associated with *Staphylococcus aureus* biofilm formation and host-pathogen interaction. *J. Bacteriol.* 195 1645–1655. 10.1128/JB.01672-12 23316041 PMC3624567

[B59] Tang H. Cao T. Liang X. Wang A. Salley S. O. McAllister J. II (2009). Influence of silicone surface roughness and hydrophobicity on adhesion and colonization of *Staphylococcus epidermidis*. *J. Biomed. Mater. Res. A* 88 454–463. 10.1002/jbm.a.31788 18306290

[B60] Turk R. Singh A. Rousseau J. Weese J. S. (2013). In vitro evaluation of DispersinB on methicillin-resistant *Staphylococcus pseudintermedius* biofilm. *Vet. Microbiol.* 166 576–579. 10.1016/j.vetmic.2013.07.011 23932310

[B61] Valle J. Toledo-Arana A. Berasain C. Ghigo J. M. Amorena B. Penadés J. R. (2003). SarA and not sigmaB is essential for biofilm development by *Staphylococcus aureus*. *Mol. Microbiol.* 48 1075–1087. 10.1046/j.1365-2958.2003.03493.x 12753197

[B62] Vergara-Irigaray M. Maira-Litran T. Merino N. Pier G. B. Penadés J. R. Lasa I. (2008). Wall teichoic acids are dispensable for anchoring the PNAG exopolysaccharide to the *Staphylococcus aureus* cell surface. *Microbiology* 154 865–877. 10.1099/mic.0.2007/013292-0 18310032 PMC2292800

[B63] Yan J. Mao J. Xie J. (2014). Bacteriophage polysaccharide depolymerases and biomedical applications. *BioDrugs* 28 265–274. 10.1007/s40259-013-0081-y 24352884

[B64] Zhang Y. Q. Ren S. X. Li H. L. Wang Y. X. Fu G. Yang J. (2003). Genome-based analysis of virulence genes in a non-biofilm-forming *Staphylococcus epidermidis* strain (ATCC 12228). *Mol. Microbiol.* 49 1577–1593. 10.1046/j.1365-2958.2003.03671.x 12950922

